# MycoperonDB: a database of computationally identified operons and transcriptional units in Mycobacteria

**DOI:** 10.1186/1471-2105-7-S5-S9

**Published:** 2006-12-18

**Authors:** Sarita Ranjan, Ranjit Kumar Gundu, Akash Ranjan

**Affiliations:** 1 Computational & Functional Genomics Group, Sun Centre of Excellence in Medical Bioinformatics, Centre for DNA Fingerprinting and Diagnostics, EMBnet India Node, Hyderabad 500076, India; 2 Bioinformatics Group, Sun Centre of Excellence in Medical Bioinformatics, Centre for DNA Fingerprinting and Diagnostics, EMBnet India Node, Hyderabad 500076, India

## Abstract

**Background:**

A key post genomics challenge is to identify how genes in an organism come together and perform physiological functions. An important first step in this direction is to identify transcriptional units, operons and regulons in a genome. Here we implement and report a strategy to computationally identify transcriptional units and operons of mycobacteria and construct a database-MycoperonDB.

**Description:**

We have predicted transcriptional units and operons in mycobacteria and organized these predictions in the form of relational database called MycoperonDB. MycoperonDB database at present consists of 18053 genes organized as 8256 predicted operons and transcriptional units from five closely related species of mycobacteria. The database further provides literature links for experimentally characterized operons. All known promoters and related information is collected, analysed and stored. It provides a user friendly interface to allow a web based navigation of transcription units and operons. The web interface provides search tools to locate transcription factor binding DNA motif upstream to various genes. The reliability of operon prediction has been assessed by comparing the predicted operons with a set of known operons.

**Conclusion:**

MycoperonDB is a publicly available structured relational database which has information about mycobacterial genes, transcriptional units and operons. We expect this database to assist molecular biologists/microbiologists in general, to hypothesize functional linkages between operonic genes *of mycobacteria*, their experimental characterization and validation. The database is freely available from our website .

## Background

Genome sequencing projects have generated large volumes of biological data which are difficult to manage and integrate effectively. This has thrown new challenges for biologists who are now supposed to decode the complex physiological information encoded within these huge genomes. A first step in this direction is to know how the various genes are organized as transcription units, operons and regulon within a genome. We have previously reported strategies and tools, such as PredictRegulon and iCR, to identify regulons in bacterial genomes and identified DtxR/IdeR associated regulons in corynebacteria and mycobacteria [1-5]. At present we are interested in developing strategies to identify transcriptional units and operons of mycobacteria.

It is well known that genes belonging to the same operon are transcribed as a single mRNA molecule in all prokaryotes. Transcription starts as the RNA polymerase binds to the promoter and continues until it reaches a transcriptional terminator. The genes of the same operon are believed to be involved in similar metabolic and physiological processes. Hence operon prediction also provides important clues to the functional relationships between the operonic genes, which can then be taken up by the experimental biologist for further validation.

A number of computational and experimental approaches are being attempted to find out which all genes are together in a genome to perform a physiological function. Among experimental approaches, RNAse Protection Assay, Dot Blot or Real Time PCR are generally used to define operon boundaries [6-9] but using these techniques for all the genes of a genome is expensive affair. A number of computational methods have been published for operon prediction [10-13] and a number of genome specific databases are also available that provide genome wide operon information [14,15].

Recently a database ODB was published [16] which has known and putative operons of many prokaryotic species including mycobacteria. However many mycobacterial transcriptional units and operons, even some known operons, are missing in this database. The advance search option requires great labor and expertise as well as external information from an average microbiologist which the latter may find difficult to provide. Therefore, there is a need to carryout more focused prediction of transcriptional units and operon in a group of related microorganisms. Such prediction and the resultant specialized database are likely to be more useful for specific research domain than global predictions. A more focused prediction in an organism also allows the researcher to revisit, track development regularly and update these databases as the research progresses in the field. Good examples of such databases are RegulonDB for *E coli*, DBTBS for *B. subtilis *and PlasmoDB for Plasmodia [15,14,17].

We present here a promising mycobacterial database MycoperonDB, which has all known data related to mycobacterial genes, including gene sequences, encoded protein sequences, known promoters, known & predicted operons and related pubmed links. These data are precomputed so that all information can be quickly accessed. The definitions of the different terms used in transcriptomics as well as one or two lines description of the important mycobacterial genes have been given on the help page as glossary. The position of different important motifs can also be searched in this database. This database will be significantly useful for the researchers working with mycobacteria. This database is an ongoing effort to increase the coverage of more and more mycobacterial species, as and when their genome sequences become available. Some of these species include *Mycobacterium smegmatis, Mycobacterium w *etc. At present, around 8256 operons are being reported in 5 mycobacterial genomes which include *M.tuberculosis H37Rv, M.tuberculosis CDC1551, M.bovis, M.avium, M.leprae*.

### Construction and contents

The overall process of transcription units and operon prediction involved multiple stages. Perl Scripts were written and used at every stage of operon prediction. These stages are-

### Retrieval of sequences

The complete genome sequences of all species of mycobacteria with original annotations were downloaded from NCBI [18].

### Orientation analysis

Genes which can be part of same operon must have same orientation. Considering this, all adjacent genes with same orientation were identified and grouped together.

### Intergenic distance analysis

Genes in an operon are often closely located on the genome as compared to those which are not in the same operon. Hence after orientation, this is another indicator to identify the operons. The intergenic distances between adjacent genes in the same orientation were calculated from the corresponding coordinates using the formula: distance_PQ _= gene Q start position – gene P end position. In general genes were passed to next operon if distance was greater than 300 bp. This cut-off was taken from *E.coli *operon prediction [19].

### Transcriptional terminators analysis

Transcription terminator site is a site where transcription terminates. Genes flanking the terminator site cannot be in the same operon. GCG Terminator program from the GCG Wisconsin software package was used to identify rho-independent transcriptional terminators. Output of GCG Terminator program was parsed for S-value >0. Finally those terminators were considered which were in the region between -20 to +200 nucleotide around the stop-codon of each mycobacterial gene of an operon (operon boundary after step 1). The genes having the terminator sites at the end were considered as end of the transcription units and operons.

### Conserved gene cluster analysis

Conserved gene clusters among genomes were identified as orthologs either on the basis of gene orders or on the basis of clusters of orthologous groups (COGs). If conserved gene clusters (adjacent genes with same orientation grouped together in more than one species) were found, then intergenic distance criteria as well as terminator criteria was relaxed, i.e. if the genes are clustered among species, they were kept in one operon.

### Integration of literature information

We scanned mycobacteria literature for reports on known transcription units, operons, promoters, and transcription start points of individual mycobacterial genes. Pubmed Id of these identified literatures was integrated with our computational prediction, for the easy and quick browsing of the articles having detailed information on promoter and operon characterization. For the published information on promoters in any one species of mycobacteria, the homologous sequences in other species were searched computationally. The search results were also incorporated in the table with the same pubmed ID.

### Development of relational database

We structured our data in the form of database. A relational database, MycoperonDB, was constructed using MySQL database management system (DBMS) to store and manage all information. MycoperonDB is currently composed of 6 tables. At present this database has information for only those mycobacterial species whose genomes are published and are available at NCBI but the same method can be used to extend the database to other genomes.

### Web Interface

In order to query the MycoperonDB database a web interface was developed using HTML, PHP, CSS and Javascript. This interface is available from our website.

### Utility and discussion

MycoperonDB aims to provide a platform to the researchers interested in mycobacteria, for a quick overview of operon and transcription unit organization of a given gene and all the related literature information like position of promoters/tsps, pubmed links, sequences of individual genes, and definition of most of the terms of mycobacterial gene regulatory circuits. A help page is also provided to guide the users step by step through the database.

### ORF search

**Figure 1:**
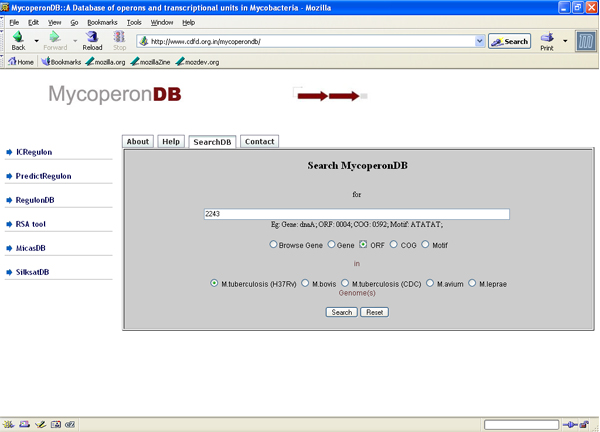
**Search form of MycoperonDB**. SearchDB is a html form which takes input from the user. The query, which can be the gene name, ORF number, COG value or motif sequence, as given in the example, should be provided in the text box. In accordance with the query, user should select the nature of the query and the species of interest by clicking appropriate radio buttons before clicking the search button.

**Figure 2:**
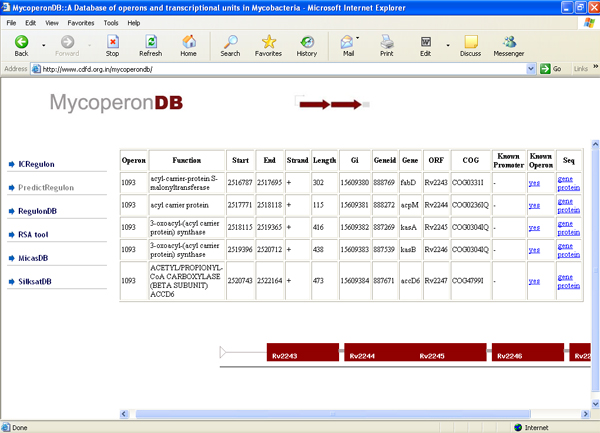
**Output page of MycoperonDB**. A typical output html page which shows the result of the user's query. The query in this case was ORF number 2243 and the species selected was *M.tuberculosis *H37Rv. The output of a search has two parts: a table and a drawing. The table shows that the query ORF is part of an operon that consists of 5 genes. The last but one column of the table shows that this is a known operon and it is hyperlinked to the relevant pubmed ID which in this case is 12464486. The last column of the table provides a quick hyperlink to gene/protein sequences of the listed operon. Each gene that is part of selected operon is drawn as maroon colored rectangle with its ORF number written on it. The drawing has a grey arrow head which depicts the forward or reverse orientation of the operonic genes on the genome.

The user can type ORF number, or gene name in the search box and the result page will show the gene cluster (if the operon has more than one gene) including the query gene with other relevant information as mentioned above (Figure 1). Separate clickable button is given for the DNA and protein sequences of the individual genes of the operon (Figure 2).

### Motif Search

The user can type any motif of interest in the search box and MycoperonDB returns the position of that motif in the whole genome. The search can be done either in one species or in more species to know the homologs of the motif across the species. If the position of the motif does not fall in the upstream region (-500 bases) of any gene, then the result page declares no operon context.

### Analyses of prediction data

We have extensively searched literature to find out the known mycobacterial operons to test how much the predictions are deviated from the actual operons. In most of the cases the predictions were in agreement with the experimental observations. For example, *mce*I operon has been shown to be transcribed as a 13 gene polycistronic message in *M. tuberculosis *[20] which is in agreement with our prediction. In our H37Rv operon table Rv0166 to Rv0178 are together. Virulence operon in *M tuberculosis *has been reported [21] and when checked in our operon table, all three genes Rv0986 to Rv0988 of this operon were found to be together. Similarly there are a number of examples like, *emb*CAB operon [22], *ini *operon [23], *mym*A operon [24], *kas*A operon [25-27] etc for which our predictions were found to be correct.

In few cases, such as *nat *operon reported in *M bovis *[28], *devR *operon, *ent *operon etc reported in *M.tuberculosis *[29,30], our prediction shows a few additional genes than reported which needs to be checked experimentally.

## Conclusion

We have predicted transcriptional units and operons in mycobacteria and organized these predictions in the form of a relational database called MycoperonDB. We further provide additional information about known and experimentally demonstrated operons, promoters and their literature links. The strengths of this database are in its simplicity, its free web accessibility, its specificity, its comprehensiveness for published mycobacterial genomes and its interactive graphical interface. This database is part of our broad effort to characterize regulons, operons and transcriptional units in mycobacteria. This database can be a practical solution for the complexity of mycobacterial genome and it is expected to assist molecular biologists as well as microbiologists dealing with mycobacteria.

## Authors' contributions

SR: Computational predictions and literature search for relevant data.

RG: Designed web page and data links.

AR: Designed web pages; designed the project and coordination.

All authors read and approved the final manuscript.
